# How to Live Long

**Published:** 1879-10

**Authors:** 


Perhaps a fair idea of the character of the book may be gathered from opening it at random, as we do now, and find on page 119, beginning with paragraph

544. He is the wisest man, and will live the longest, who makes business a pleasure, and the acquisition of money an ambition, as a means of benefitting and elevating and blessing others.

545. The ability to make money is a talent; the ability to keep it is two; the ability to use it wisely and well, is ten.

546. As the world gets older and more crowded, the rivalries of life become greater, the strife for bread more trying, the rage for riches more reckless; and in all cases, those who insist on being foremost, will enjoy life least, and soonest lose it.

547. Hurry is the bane of modern civilization, and is often blind.

548. The busiest man sometimes breaks the soonest.

548. "Let alone" is a grandly effectual remedy in very many of the accidents and diseases to which humanity is liable; it is the universal remedy of the animal creation.

550. A single principle admits, sometimes, of a dozen different applications in connection with the well-being of the body.

551. It is a favorite saying, and considered wise by all, "Take things by their smooth handle;" and it might be a source of a greater amount of human enjoyment, if the average motto in reference to the occupations of life were,

"Choose the Easiest, for there is no virtue in aiming to accomplish difficult things, simply because they are so." The two or three pages of paragraphs in reference to married life, beginning with 1,252, "A good wife is the greatest earthly blessing," may be read to supreme advantage once a week for a life-time by all married persons; for example 1,258, "Never both be angry at once;" there are forty sermons in that text, a bonanza of incalculable value, a precept which every one can follow. "You can't open it at any page without finding something in it worth five dollars, if you didn't know it before.

Amusing incidents and narrations are scattered through the book, as a means of waking up the sleepy, and arousing the attention to important truths near at hand. Among the lessons and sentiments inculcated, are—

Work by the day, and not by the job, if you want to be healthy.

Those who know most, seldom make positive statements on any subject.

"Beverages" are tempters to the first steps towards drunkenness.

The almost universal cause of dyspepsia, is eating too often, too fast, and too much.

If you must strike your child, let it be after mature deliberation.

An encouraging word, or a cheerful look, often does the patient more good than pill, powder or potion.

Neither poverty, hardships, nor exposure, tend to lengthen life, but shorten it many years.

While women should not marry under twenty, men should wait until twenty-five.

Such are some of the sentiments found in the book on opening its pages at random. The ideas are not original with the author, for truth is eternal, but the language is, in all cases.

ENCOURAGING.—Halifax commercial papers complain that since the stringent temperance laws were passed by the Nova Scotia legislature, the trade in liquors is very dull; that only 195 licenses were taken in that city, a reduction of 92 since last year, and that the city revenue is suffering in consequence, to the amount of several thousand dollars. They may comfort themselves with the prospect that their criminal expenses will be correspondingly reduced.

